# Clinical Significance of Numerous Irregular Polypoidal Lesions in the Duodenum

**DOI:** 10.4103/1319-3767.61246

**Published:** 2010-04

**Authors:** Ahmed Helmy, Lina Asaad, Ingvar Kagevi, Hamad I. Al-Ashgar

**Affiliations:** Department of Medicine, King Faisal Specialist Hospital and Research Center, Riyadh, Saudi Arabia; 1 Department of Pathology, King Faisal Specialist Hospital and Research Center, Riyadh, Saudi Arabia

The patient, a 20-year-old obese male, complained of epigastric pain of two years duration with no associated nausea or vomiting. He underwent upper gastrointestinal endoscopy in a local hospital and received treatment for *H. pylori* infection, with no significant improvement. He was referred to King Faisal Specialist Hospital and Research Centre (KFSHRC) for further evaluation. Laboratory investigations showed: WBCs: 7.3×10^9^/L. Hb: 133 g/L. MCV: 69.6 fL. Platelets: 500×10^9^/L. ESR: 45 mm/hour. CRP: 10.8 mg/L. Urea: 4.0 mmol/L. Creatinine: 72 umol/L. K^+^: 3.9 mmol/L. Na^+^: 140 mmol/L. Cl-:103 mmol/L. CO_2_: 22 mmol/L. Albumin: 44 g/L. Bilirubin: 8 umol/L. LD: 177 U/L. ALT: 108 U/L. AST: 61 U/L. ALP: 64 U/L. GGT: 161 IU/L. Negative antinuclear antibody screen as well as celiac disease serology.

Follow-up endoscopy after six months showed normal esophageal mucosa, nodular congested gastric mucosa and nodular duodenal mucosa with variable-sized polypoid lesions [Figures 1a and b], which were biopsied and sent for pathological examination [Figure 2 a–c].

**Figure 1:**
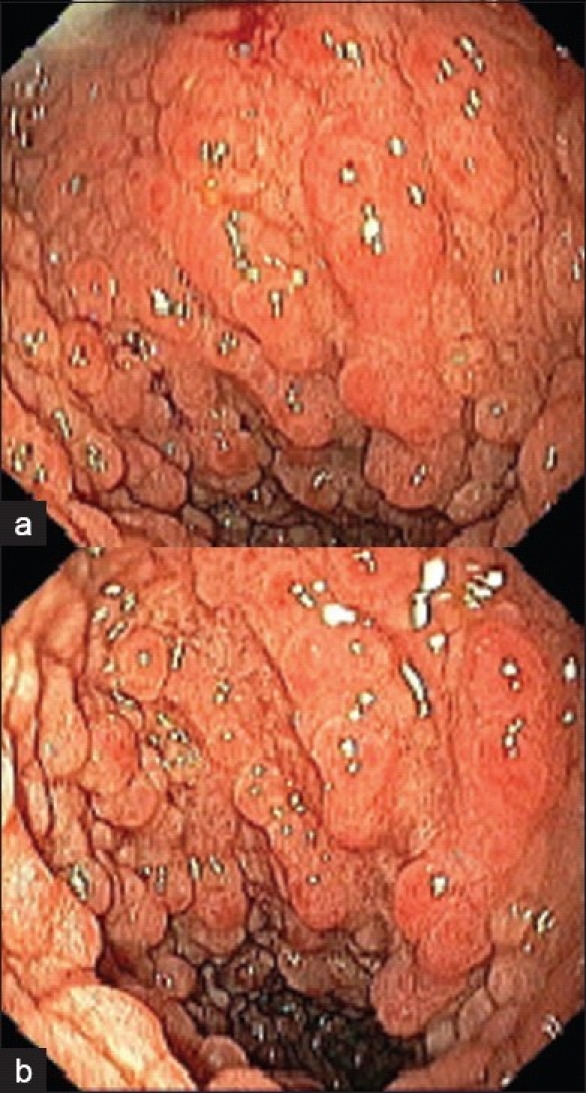
(a, b)

**Figure 2:**
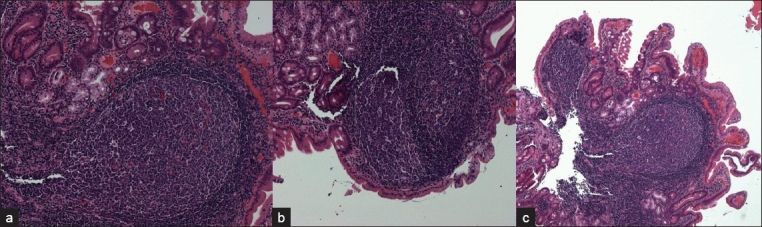
(a, b, c)

## QUESTIONS

Q1. What is the diagnosis?

Q2. What are the histopathological findings of duodenal polypoidal lesions?

Q3. What other sites can be affected by this pathology?

Q4. What is the clinical significance of this abnormality?

## ANSWERS

A1. The diagnosis: Reactive follicular lymphoid hyperplasia (FLH).

A2. Histopathological examination of the first part of duodenum polypoidal lesions biopsies showed focal villous atrophy with prominent reactive FLH in the lamina propria with flattened overlying mucosa, and no evidence of intraepithelial lymphocytes, dysplasia, or neoplasia [Figures 2a–c]. Gastric antral biopsy showed moderate chronic active gastritis, and Giemsa stain showed the presence of *H. pylori* organism.

A3. FLH can develop wherever lymphoid tissue is present. The mostly reported sites of FLH include hard palate and oral cavity, entire gastrointestinal tract, nasopharynx, larynx, bronchi, parotid gland, breasts, skin, spleen, peripheral nerves, and the thymus gland.

A4. FLH is an uncommon benign proliferation of lymphoid follicles, a poorly understood entity, which may be confused clinically and histologically with malignant lymphoma. It has been alternatively named benign lymphoid hyperplasia, reactive lymphoid hyperplasia, and pseudolymphoma. The cause of FLH is unknown, but may be associated with common variable hypogammaglobulinemia, primary immunodeficiency states and Epistein Barr virus infection. The course of FLH in children is benign, but the outcome in adults is controversial. Histopathologic examination, immunohistochemical analysis and molecular studies are essential to achieve accurate diagnosis and to implement appropriate management. The patient under study was given *H. pylori* eradication therapy, and reassured.

